# How the COVID-19 pandemic effected economic, social, political, and cultural factors: A lesson from Iran

**DOI:** 10.1177/0020764020939984

**Published:** 2020-07-02

**Authors:** Javad Yoosefi Lebni, Jaffar Abbas, Farideh Moradi, Mohammad Reza Salahshoor, Fakhreddin Chaboksavar, Seyed Fahim Irandoost, Nazila Nezhaddadgar, Arash Ziapour

**Affiliations:** 1 Health Education and Health Promotion, Health Institute, Kermanshah University of Medical Sciences, Kermanshah, Iran; 2 Antai College of Economics and Management and School of Media and Communication, Shanghai Jiao Tong University, Shanghai, China; 3 Clinical Research Development Center, Imam Reza Hospital, Kermanshah University of Medical Sciences, Kermanshah, Iran; 4 Life Style Modification Research Center, Imam Reza Hospital, Kermanshah University of Medical Sciences, Kermanshah, Iran; 5 Department of Anatomical Sciences, Medical School, Kermanshah University of Medical Sciences, Kermanshah, Iran; 6 Health Education and Health Promotion, Department of Public Health, School of Health, Urmia University of Medical Sciences, Urmia, Iran; 7 Department of Health Care Services and Health Education, School of Health, Ardabil University of Medical Science, Ardabil, Iran

The members of the coronavirus (CoV) family refer to infectious disease, which refer to CoV flu, CoV pneumonia, or a respiratory syndrome. COVID-19 causes various contagious diseases, such as the Middle East respiratory syndrome (MERS), severe acute respiratory syndrome (SARS), which causes common strains of the cold. CoVs are of the new diseases of the respiratory system (Chen et al., 2020), which is a subset of the sizable Coronaviridae family, belonging to the genus *Nidovirales* (Plant et al., 2013). The term “coronavirus” originates from the Latin word “corona,” which means crown or halo (Hindson, 2020). The corona word refers to the appearance of virions (the infectious form of the virus), as is seen under an electron microscope. This virus has a large, onion-like margin and is reminiscent of a picture of a royal crown or solar crown. Hence, CoV refers to “crowned virus” as well (Shuja et al., 2020).

The outbreak of the infectious virus first time appeared in Wuhan, China, in late December, 2019, and later this pandemic (COVID-19) rapidly spread in other cities of China and over 200 countries worldwide by end of 25 April 2020 (Chen et al., 2020). The Irani Government officially confirmed the attack of COVID-19 in Iran on 18 February 2020 and imposed suppression and lockdown in all the provinces, which remains the key focus of this article. This study covers the critical challenges because of CoV transmission, such as, economic, social, cultural, legal, and political factors.

Economic factors: the financial inability of the government of Iran to protect the nation financially during quarantine was not sufficient, which in turn caused the majority of people, who usually wing their bread, and remain active in many crowded places and business centers. It made a descendant course of the COVID-19 infectious disease and its control impossible as social distancing, which is one of the most critical principles to reduce the risk of contracting the virus, specified a disruption (Yezli & Khan, 2020). Besides, considering the current economic recession of Iran, allocating a portion of the budget to deal with the current crisis could lead the country to a crisis in the supply chain of the resources needed by the people, further reduction in foreign exchange resources, unemployment, and higher inflation. Among the other critical elements in this category can be the credit limit of the health sector to supply medical equipment (Abbas et al., 2018).

Social factors: although most people observed self-quarantine at homes; however, it developed empathy and intimacy among family members at a larger scale. These social factors caused psychological problems and violence by creating differences among people as well as verbal and physical disputes. Besides, staying at home for a long time may lead to depression, lethargy, and exacerbation of previous illnesses like hypertension, stress, and anxiety. The other social factor refers to the lack of adequate consumption of antiseptics at home, which leads to other problems, such as damage to the respiratory system. People have reacted to this pandemic and became sensitive in interacting other individuals at large (Paital et al., 2020).

Cultural factors: it becomes necessary to abuse certain social conditions and to endanger the safety and health of people by some people, leading to hoarding and smuggling of basic health products and food (Abbas, Aqeel, Jaffar et al., 2019).

Legal factors: the lack of participation of the private sector in the performance of semi-clinical services (laboratory and radiology) in the outpatient unit and inpatient wards of patients with COVID-19 in the hospital indicates that there are no clear and specific rules in this area (Mubeen et al., 2020). Besides, the improper and unprincipled use of disinfectants by the public in the city and the lack of proper supervision in doing so by authorized persons, as well as harming the environment, destroy public property, such as machines, and ATM cards (Abbas, Aman et al., 2019).

Political factors: although clerics have cooperated in advising communities to remain at homes and follow lockdown policy of the government and performed religious obligations at homes, such as Friday prayers. Besides, people did not practice a suppression strategy, and the government did not suspend visits to pilgrimage locations, which was a contradicted policy of the Irani government. It caused a rapid spread of the COVID-19 in all provinces in Iran because of lacking control over disease transmission. It is vital to note that this issue was first limited, then managed and controlled. The other challenge was to diagnose patients with the disease and whose polymerase chain reaction (PCR) tests indicated virus-positive, they were considered as halo, and those with symptoms were not reported on computed tomography (CT) scans for it, which in turn led to a discrepancy between official and actual reports (Abbas, Mahmood et al., 2019; Paital et al., 2020).

As mentioned above, the factors directly correlate with the mental health of ordinary individuals from one end of the disease; however, there is a severe fear that thousands of deaths are from the other end. The COVID-19 outbreak has adversely affected the economy, and people have lost their jobs, which has failed in gaining a minimum income level to run livelihood. The fear of economic loss has increased stress and leads to mental health problems among people worldwide.

The government of Iran has taken protective measures and policies to reduce anxiety among the public to tackle mental health problems. The government of Iran established specialized health centers to treat CoV patients after discharge from the leading hospitals. This motive was helpful to keep COVID-19 patients separate from their families until the full recovery, which was beneficial to prevent further spread of the deadly disease. The government offered utility bills delay for 3 months, and payment in installments was helpful to provide comfort to patients' families, which reduced mental stress.

The officials paid full attention to reduce anxiety, stress, and mental health issues by providing necessary measures. The government has initiated schemes to encourage support donors who cooperated with poor and low-income individuals with financial support. Online and telephonic psychological counseling services to cater to CoV patients and other healthy people are useful through free-of-cost initiative of the government. It has helped to reduce the community’s mental stress. The government has started loans at the low-interest rate for needy people, controlled social network sites to identify and prevent spreading sensational and false information about CoV disease. It helped to manage the terror of fake facts about COVID-19 in the community.

The government launched social support programs, and psychological protocol services for the survivors of COVID-19 through screening, and identifying suspects of this disease with mental health surveys. Plans were launched Covid-19 dissemination in the cyberspace, education, and correct informing was provided to the mass public through media to prevent fear and terror in the society. The government launched special services for CoV patients for the funeral process as it is one of the cultural factors in society. The government launched programs to educate the public about the spread of CoV disease and reduce the public mental stress in the community. The protective measures helped to build confidence among people, which helped to overcome fear, anxiety, stress, and mental health problems.

The health experts recommended solutions to slow down, sustain and overcome the transmission of COVID-19 problems as mention below:

- Priority for investment in the health sector compared to other areas such as military, weapons, and armaments.
- Allocation of subsidies to all citizens with private business, low-interest loans to large and small medium enterprises.
- Specific attention to vulnerable and elderly people and actively diagnosis of the disease.
- A comprehensive view of the health sector and the participation of all agencies and institutions such as the government and non-governmental sectors.

## References

[bibr1-0020764020939984] Abbas J. Aman J. Nurunnabi M. Bano S. (2019). The impact of social media on learning behavior for sustainable education: Evidence of students from selected universities in Pakistan. Sustainability, 11(6), 1683–1689.

[bibr2-0020764020939984] Abbas J. Aqeel M. Jaffar A. Nurunnabi M. Bano S. (2019). Tinnitus perception mediates the relationship between physiological and psychological problems among patients. Journal of Experimental Psychopathology. Advance online publication. 10.1177/2043808719858559

[bibr3-0020764020939984] Abbas J. Aqeel M. Wenhong Z. Aman J. Zahra F. (2018). The moderating role of gender inequality and age among emotional intelligence, homesickness and development of mood swings in university students. International Journal of Human Rights in Healthcare, 11(5), 356–367. 10.1108/ijhrh-11-2017-0071

[bibr4-0020764020939984] Abbas J. Mahmood S. Ali H. Ali Raza M. Ali G. Aman J. Nurunnabi M. (2019). The effects of corporate social responsibility practices and environmental factors through a moderating role of social media marketing on sustainable performance of business firms. Sustainability, 11(12), 3434–3439.

[bibr5-0020764020939984] Chen S. Yang J. Yang W. Wang C. Bärnighausen T. (2020). COVID-19 control in China during mass population movements at New Year. The Lancet, 395(10226), 764–766. 10.1016/s0140-6736(20)30421-9 PMC7159085 32105609

[bibr6-0020764020939984] Hindson J. (2020). COVID-19: Faecal–oral transmission? Nature Reviews Gastroenterology & Hepatology, 17(5), 259. 32214231 10.1038/s41575-020-0295-7 PMC7095230

[bibr7-0020764020939984] Mubeen R. Han D. Abbas J. Hussain I. (2020). The effects of market competition, capital structure, and CEO duality on firm performance: A mediation analysis by incorporating the GMM model technique. Sustainability, 12(8), 3480–3486.

[bibr8-0020764020939984] Paital B. Das K. Parida S. K. (2020). Inter nation social lockdown versus medical care against COVID-19, a mild environmental insight with special reference to India. Science of the Total Environment, 728, 138914. 10.1016/j.scitotenv.2020.138914 PMC7179495 32339832

[bibr9-0020764020939984] Plant E. P. Sims A. C. Baric R. S. Dinman J. D. Taylor D. R. (2013). Altering SARS coronavirus frameshift efficiency affects genomic and subgenomic RNA production. Viruses, 5(1), 279–294. 10.3390/v5010279 23334702 PMC3564121

[bibr10-0020764020939984] Shuja K. H. Aqeel M. Jaffar A. Ahmed A. (2020). COVID-19 pandemic and impending global mental health implications. Psychiatria Danubina, 32(1), 32–35. 10.24869/psyd.2020.32 32303027

[bibr11-0020764020939984] Yezli S. Khan A. (2020). COVID-19 social distancing in the Kingdom of Saudi Arabia: Bold measures in the face of political, economic, social and religious challenges. Travel Medicine and Infectious Disease. Advance online publication. 10.1016/j.tmaid.2020.101692 PMC7172679 32330561

